# Effects of Functional Interactivity on Patients’ Knowledge, Empowerment, and Health Outcomes: An Experimental Model-Driven Evaluation of a Web-Based Intervention

**DOI:** 10.2196/jmir.1953

**Published:** 2012-07-18

**Authors:** Luca Camerini, Peter Johannes Schulz

**Affiliations:** ^1^Institute of Communication and HealthFaculty of Communication SciencesUniversità della Svizzera italianaLuganoSwitzerland

**Keywords:** Interactivity, health literacy, patient empowerment, fibromyalgia, Internet intervention

## Abstract

**Background:**

The effectiveness of eHealth interventions in terms of reach and outcomes is now well documented. However, there is a need to understand not only whether eHealth interventions work, but also what kind of functions and mechanisms enhance their effectiveness. The present investigation contributes to tackling these challenges by investigating the role played by functional interactivity on patients’ knowledge, empowerment, and health outcomes.

**Objectives:**

To test whether health knowledge and empowerment mediate a possible relationship between the availability of interactive features on an eHealth application and individuals’ health outcomes. We present an empirical, model-driven evaluation of the effects of functional interactivity implemented in an eHealth application, based on a brief theoretical review of the constructs of interactivity, health knowledge, empowerment, and health outcomes. We merged these constructs into a theoretical model of interactivity effects that we tested on an eHealth application for patients with fibromyalgia syndrome (FMS).

**Methods:**

This study used a pretest–posttest experimental design. We recruited 165 patients and randomly assigned them to three study groups, corresponding to different levels of functional interactivity. Eligibility to participate in the study required that patients (1) be fluent in Italian, (2) have access to the Internet, (3) report confidence in how to use a computer, and (4) have received a diagnosis of FMS from a doctor. We used structural equation modeling techniques to analyze changes between the pretest and the posttest results.

**Results:**

The main finding was that functional interactivity had no impact on empowerment dimensions, nor direct observable effects on knowledge. However, knowledge positively affected health outcomes (b = –.12, *P *= .02), as did the empowerment dimensions of meaning (b = –.49, *P *< .001) and impact (b = –.25, *P *< .001).

**Conclusion:**

The theoretical model was partially confirmed, but only as far as the effects of knowledge and empowerment were concerned. The differential effect of interactive functions was by far weaker than expected. The strong impact of knowledge and empowerment on health outcomes suggests that these constructs should be targeted and enhanced by eHealth applications.

## Introduction

The effectiveness of eHealth interventions in terms of reach and outcomes is now well documented. Several reviews and meta-analyses showed the benefits of designing, implementing, delivering, and maintaining health programs on the Internet [1-7]. Still, Bennett and Glasgow [8] pointed out that one of the major challenges for eHealth research is to evaluate the differential effects of the enabling functions implemented in the applications, rather than consider them as black boxes [8]. In other words, there is a need not only to know *whether *eHealth interventions work, but also to understand *how *they achieve their effectiveness.

The question of how eHealth interventions work has at least two aspects. The first is to identify the effective elements in these interventions; this comes down to achieving a more precise understanding of the independent variable. As it is among the prime features that distinguish Internet interventions from other mediated communication interventions, an obvious subject to turn to is the role played by interactivity for the outcome of eHealth interventions. Indeed, in the health care domain, interactive applications are favored, and optimistic claims on their effectiveness abound [9-16]. However, the body of evidence on the effects of interactivity on behavioral and health outcomes is not very broad. This is perhaps linked to the fact that the definition and operationalization of interactivity have always been difficult [15]. According to Rafaeli and Ariel [15] and Rafaeli [17], a rough distinction can be made between the conceptualizations focused on functions of features and those focused on users.

Interactivity as an attribute of technology (eg, [18]) can be defined as “the extent to which users can participate in modifying the form and content of a mediated environment in real time” [19,20]. In this sense, traditional media are low in terms of interactivity, while new technologies, such as the Internet, share a high level of interactivity. This perspective is known as functional interactivity and offers a theoretically robust way to operationalize an eHealth intervention in terms of its enabling functions.

The second aspect is to better understand the process that creates the effect. This means to find intervening variables that mediate or moderate the effect of eHealth interventions. As eHealth interventions mostly aim at improving self-management and self-help capabilities, approaches from the larger field of patient autonomy come into focus: health literacy (or rather one of its elements, patient knowledge) and patient or health empowerment. Both are assumed or have been shown to affect health outcomes.

In its original definition, health literacy was conceived as “the ability to read and comprehend prescription bottles, appointment slips, and other essential health-related material” [21]. Reading and numeracy are considered essential skills an individual should possess in order to navigate the health care system [22,23]. This functional perspective, however, has been criticized for being too narrow and missing much of the richness of the deeper meaning and purpose of literacy for people (eg, [24]). As a consequence, broader definitions have been proposed [24,25]. For this study, we adopted the critical perspective on health literacy and health knowledge proposed by Schulz and Nakamoto [26]. These authors conceived health literacy and knowledge as a function of basic reading and writing skills, declarative knowledge, procedural knowledge, and judgmental skills.

Patient or health empowerment, in turn, was theorized as the process by which people, organizations, and communities gain mastery over issues of concern to them [27-39]. Thomas and Velthouse [40], building on the psychological and organizational literature [41-43], proposed a model of empowerment as a multidimensional construct, composed of four cognitive variables (or task assessments): impact (or the degree to which behavior is seen as “making a difference”), competence (or the degree to which a person can perform task activities skillfully), meaningfulness (or the individual’s intrinsic caring about a given task), and choice (or weather a person’s behavior is perceived as self-determined). In the health care domain, powerlessness has been associated with ill health [44], whereas empowerment is considered a determinant of improved health status [45-47] and eHealth interventions, especially online support groups, are considered an effective empowering tool [48-50]. A recent review and meta-analysis of the effectiveness of Web-based interventions on patient empowerment showed that eHealth significantly contributes to increased empowerment, even when compared with face-to-face consultations or usual care [51].

Links between eHealth interactivity on the one hand, and health literacy and empowerment on the other, are plausible to assume, for it takes knowledge and literacy to benefit from eHealth applications, and bringing them up in medical consultation gives a more active role to patients, who thus claim autonomy. Knowledge and empowerment can also be assumed to be affected by eHealth interventions and their qualities. In a sense, such links are obvious to assume, but they are largely unexplored by research and therefore not established at all [52]. Our research aimed at contributing to this area, resting on the assumption that eHealth fosters patient autonomy. In particular, we hypothesized that the availability of interactive functions in eHealth interventions would positively affect users’ knowledge and their empowerment, and further that knowledge and empowerment would positively affect health outcomes.

**Figure 1:**
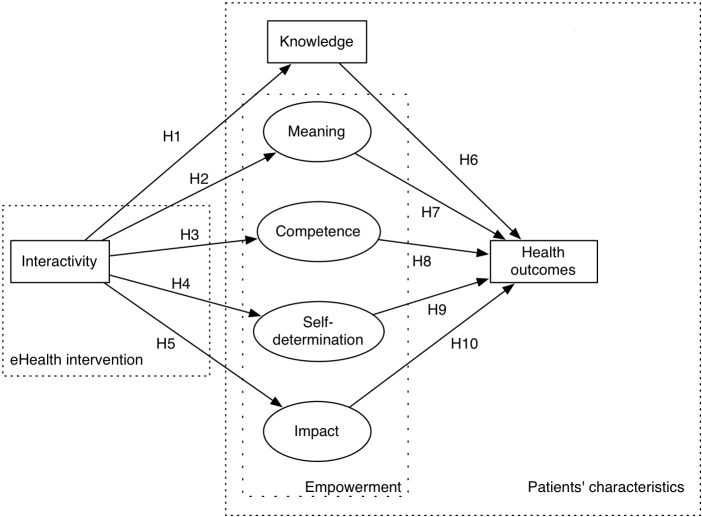
A model of functional interactivity effects on knowledge, empowerment, and health outcomes. Arrows indicate directional effects. H1-H10 = main hypotheses.

The main aim of this study was to test whether health knowledge and empowerment mediate a possible relationship between the availability of interactive features on an eHealth application and individuals’ health outcomes. We expected that interactivity increases knowledge and empowerment, which in turn improve patient assessment of fibromyalgia impact, our health outcome measure. Similar assumptions for other interventions and conditions were already tested—and partially confirmed—in other studies relating interactive eHealth applications with knowledge or empowerment, or both [10,53-56]. Therefore, this study tested a specific model that conceives aspects of patient autonomy as mediating a possible influence of interactivity on assessment of impact. To keep a confirmatory rather than an exploratory approach to these relationships, we did not directly test alternative models (eg, that knowledge and empowerment could also moderate the effect of interactivity). Figure 1 illustrates our model of interactivity effects on patients’ assessment of fibromyalgia impact.

Each arrow in Figure 1 represents a hypothesis, all of which are a specification of the general mediator hypothesis mentioned.

Specifically, patients who use an application that offers interactive functions become more knowledgeable (H1) and achieve a higher score on the empowerment dimensions of meaning (H2), competence (H3), self-determination (H4), and impact (H5) than patients not offered these functions. In turn, the higher the level of knowledge (H6), meaning (H7), competence (H8), self-determination (H9), and impact (H10), the better (ie, lower) the patients assess their fibromyalgia impact.

The mediator analysis rests on the existence of an influence of interactivity condition on outcome, in the sense that patients who are offered interactive function will assess fibromyalgia impact better. We also tested this.

## Methods

### Reference Population

The reference population in this study consisted of patients with fibromyalgia syndrome (FMS). According to the American College of Rheumatology [57], FMS is a condition characterized by chronic widespread pain and tenderness in 11 or more of the 18 specific tender point sites. Although the medical evidence is still lacking for precise diagnostic criteria for FMS, there are three major symptoms that are usually associated with the disease: pain, sleep disorders, and fatigue [58-60]. Alongside these somatic factors are many other psychological dimensions observed in FMS patients, such as anxiety, stress, and depression [61-63]. People with FMS usually have other concurrent conditions, including diabetes, high blood pressure, and back pain. FMS outcomes are generally measured with the Fibromyalgia Impact Questionnaire (FIQ) [64-66] or the Fibromyalgia Assessment Status (FAS) [67]. The FIQ includes items covering all the disease-specific domains, accounting for functional disability, job ability, pain intensity, sleep function, stiffness, anxiety, depression, and the overall sense of wellbeing. The FAS focuses on the three main characteristics of FMS: fatigue, sleep disturbances, and pain. Both of these measures have good psychometric properties, in terms of reliability and validity. For this study, we used the FIQ, as the FAS was still under development and testing. In other words, our health outcome variable was patients’ assessment of fibromyalgia impact.

### The eHealth Intervention

To test our hypotheses, we developed a Web-based eHealth intervention for patients with FMS. This application, called ONESELF, was developed in collaboration with health professionals (rheumatologists, physiotherapists, and general practitioners), and it is fully compliant with the Health On the Net Foundation guidelines (HONcode). The HONcode prescribes guidelines on the quality of the contents and the overall usability of an application. Indeed, these intrinsic factors can play a decisive role in the ultimate effects of an online intervention, and compliance with these guidelines helps to temper—and to some extent rule out—potential biases caused by usability issues.

The application enabled asynchronous and synchronous interactions with health professionals and laypeople. The system also included static informative sections in a virtual library that provided users with relevant information on the disease. First aid and frequently asked questions sections provided brief and practical information on syndrome management. A virtual gymnasium provided patients with tailored multimedia contents on several physical exercises that constitute the wider part of the nonpharmacological treatment of FMS. Later, a section with testimonies, where patients could post their stories and read stories of other people with the same health condition, enhanced the dimension of social support. We also designed and implemented synchronous interaction via a chat room and asynchronous interaction in an online forum. Patients used these tools to communicate with the physicians and among themselves. A more detailed description of the design of the application is presented elsewhere [68,69], together with qualitative insights on the user experience with the system, which was generally considered useful, usable, and comprehensible. Since the first release of ONESELF in June 2008, more than 600 FMS patients, mostly from Switzerland and Italy (the site language being Italian), have registered.

### Study Design and Procedure

**Figure 2:**
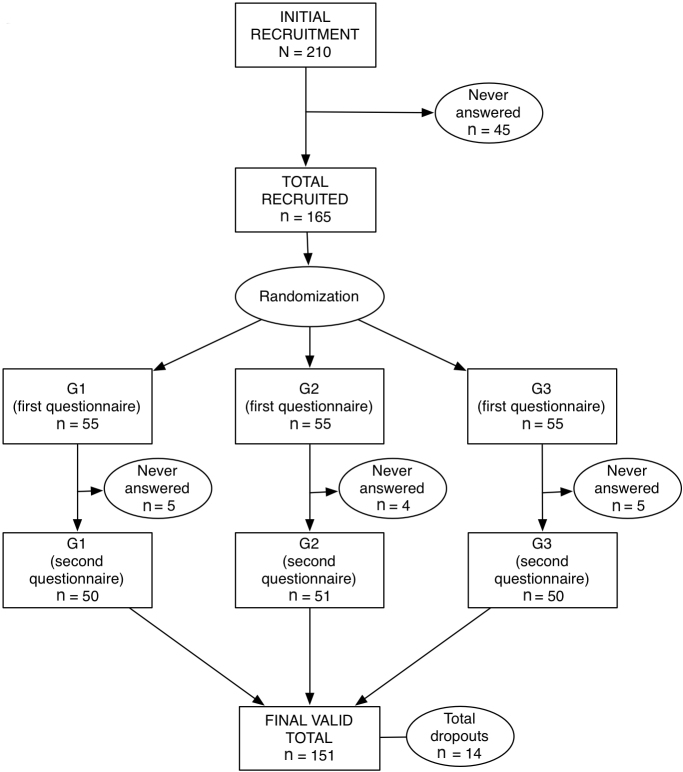
Attrition flow from the initial recruitment phase. G1 = static-only study group; G2 = interactive-only study group; G3 = full application study group.

This study used a pretest–posttest experimental design. Patients were contacted by two means: a list of patients who were members of the *Associazione Fibromialgici Svizzeri Sezione Ticino *(Ticino Fibromyalgia Patients Association) and patients visiting health professionals (rheumatologists and physiotherapists). Health professionals were involved in the recruitment to assure that patients received a diagnosis of FMS from a doctor. To be eligible for the study, patients had to meet a set of inclusion criteria: (1) be fluent in Italian, (2) have access to the Internet, (3) be confident in using a computer, and (4) have received a diagnosis of FMS from a doctor. Every patient who matched these criteria was given a letter briefly describing the aims of the study, together with a contact form. If interested in the study, patients had to send the form back to the research team, filled in with contact details. The research team contacted the patients by phone and email. Patients were introduced to the study and asked to register on the ONESELF website. After registration, they had to accept an informed consent statement and, finally, complete the first questionnaire. After completing the questionnaire, patients could access the website and start the navigation. After 5 months of access to the site, a second questionnaire was presented and completed. A maximum of three reminders were used to maximize response rate. By the end of the recruitment process, a total of 165 patients had agreed to participate in the study (Figure 2).

To investigate the effect of functional interactivity, we created three different versions of the ONESELF application, each implementing different enabling functions. Patients were randomly assigned to one of the three versions and blinded to the others, using a computer utility that assigned them to a randomly selected experimental condition until the conditions were equally filled. Patients in group 1 (n = 55) were given a static version of ONESELF, including only the library, the virtual gymnasium, the testimonials, and the generic sections such as the first aid, the frequently asked questions, and other common contents (eg, contacts, legal notices, and ownership disclosure). No interactive enabling tools such as the Web forum or the chat room were present in this version. This group was considered as the reference or control group. Patients in group 2 (n = 55) were given an interactive-only version of ONESELF, including the Web forum, the chat room, and the generic sections. Static sections were not implemented in this version. Patients in group 3 (n = 55) were given the full version of ONESELF, including both static and interactive components.

The rationale behind this choice goes back to the functional approach to interactivity. While the library, the virtual gymnasium, and the testimonials did not allow any input from the user other than traditional hypertextual navigation, the Web forum and the chat room enabled synchronous and asynchronous interactions. Although this kind of approach has been criticized for being too generic [15], it is useful to capture the contribution of different enabling functions [70], and it was adopted in other studies on interactivity as well (eg, [53,71-73]).

### Measurements

Each construct of the eHealth intervention effect model was translated into operational measures. The assessments were conducted using standardized online questionnaires. The questionnaires were pretested for face and content validity with two focus groups, with 4 health professionals and 4 patients respectively. The measures used in the study were as follows.

#### Demographic Characteristics

Demographics in the questionnaires included age, gender, and level of education. We used age as an exogenous covariate in the model analysis, since it can discriminate other focal constructs (eg, level of pain varies with age). Gender was measured but was not controlled for, since the large majority of FMS patients are female and our sample reflected this [74].

#### Knowledge

Knowledge was measured following the approach of critical or integrative models of health literacy. Thus, we assessed knowledge with 10 multiple choice questions adapted from the Mayo Clinic website relating to FMS symptoms, etiology, treatments, and management. Each answer was coded 1 when correct and 0 when incorrect. The final measure of knowledge was obtained by a mean score calculation of the 10 items, with a theoretical range from 0 (no correct responses) to 1 (all correct responses).

#### Empowerment

Empowerment was measured according to the scale proposed by Spreitzer [75] but adapted to the FMS domain. This measure reflects the multidimensionality of the construct of empowerment, which is a combination of meaning, competence, self-determination, and impact. Each one of the subdimensions is treated as a latent construct with three observed indicators. Each indicator was measured on a 7-point Likert scale.

#### Health Outcomes

Health outcomes were measured with the FIQ [64,65] in its Italian version [76]. The FIQ is a validated questionnaire that consists of 20 indicators to assess patients’ disability to carry out everyday activities, patients’ intensity of pain, and the interference of FMS with patients’ sleep and emotional state. The FIQ provides a single score ranging from 0 to 100, where a higher score indicates a greater impact of FMS on the patient. For this reason, it should be considered a measure of negative health outcomes. According to Bennett [65], the average FMS patient scores about 50. Because of the high theoretical variance of this measure compared with the others’, we transformed the FIQ raw score on a 0–10 scale and used the result as a single manifest indicator throughout the analyses.

#### Additional Covariates

The number of years since the first diagnosis was measured as a single additional covariate. By doing so, it is possible to control for potential differences that are due to the illness experience accumulated over time.

In addition to this set of self-report measures, we operationalized functional interactivity by delivering different versions of ONESELF to the three groups of patients, as explained above.

### Analyses

The main analyses were conducted using structural equation modeling techniques [77]. SPSS AMOS 18 (IBM Corporation, Somers, NY, USA) was used for the analyses. Specifically, we examined the effect of interactivity on knowledge, meaning, competence, self-determination, and impact. Because of the relatively small sample size, it was not advisable to run a test on a model including the five focal dependent variables at the same time. Thus, a structural equation model reflecting an analysis of covariance for the analysis of change was implemented and tested five times, varying the five focal dependent variables. Additionally, to determine whether a mediating effect existed, we ran an analysis of variance with the fibromyalgia impact as dependent variable and study condition as factor.

The model accounts for autoregressive effects of the focal dependent variables (knowledge and the four empowerment dimensions) at time 1 on the same variable at time 2, as well as outcomes at time 1 on outcomes at time 2. This strategy allows controlling for pretest scores and adjustment for measurement errors. To reduce sampling error, we included age and years since first diagnosis in the model as additional covariates, as well as the initial score on health outcomes. The main endogenous variable was the patients’ assessment of fibromyalgia impact.

## Results

### Sample Characteristics and Application Usage

The sample included 165 patients with FMS. Age ranged from 27 to 72 (mean 49.93, SD 9.93) years. Most patients were female (157, 95.2% women; 8, 4.8% men). The modal educational level was high school (63.6%); 43 (26.1%) had a lower educational level and 17 (10.3%), a higher. The mean time since first diagnosis was 5.7 (SD 4.8) years. At baseline, the average FIQ score was 5.98 (SD 1.82). Table 1 presents descriptive sample characteristics.

Table 2 reports the mean values of the relevant constructs of the model, as measured at pretest (T1) and posttest (T2).

**Table 1:** Descriptive sample characteristics.

Categorical or dichotomous variable		Range	n	%
**Gender, n (%)**				
	Female		157	95.2%
	Male		8	4.8%
**Level of education, n (%)**				
	None		4	2.4%
	Elementary school		9	5.5%
	Middle school		30	18.2%
	High school		105	63.6%
	University		17	10.3%
**Continuous variables, mean (SD)**			**Mean**	**SD**
	Age (years)	27–72	49.93	9.93
	Years since first diagnosis	0–40	5.7	4.8
	Health outcomes (FIQ^a^) at baseline	0.73–9.07	5.98	1.82

^a ^Fibromyalgia Impact Questionnaire, score transformed on a scale of 0 to 10.

**Table 2:** Descriptive statistics (mean, SD) for experimental measures for study groups 1–3 at time 1 (pretest) and time 2 (posttest)^a^.

Variable name	Time 1			Time 2		
	Group 1 (n = 55)	Group 2 (n = 55)	Group 3 (n = 55)	Group 1 (n = 50)	Group 2 (n = 51)	Group 3 (n = 50)
Health outcomes	5.9 (1.7)	6.1 (1.7)	5.9 (1.8)	5.9 (1.7)	6.0 (1.5)	5.8 (1.8)
Knowledge	6.1 (1.8)	5.6 (1.9)	5.9 (1.6)	6.4 (1.9)	6.1 (1.7)	6.4 (1.6)
Meaning-1	6.3 (1.4)	6.4 (1.3)	6.5 (0.9)	6.6 (0.8)	6.3 (1.3)	6.4 (1.0)
Meaning-2	6.0 (1.6)	6.1 (1.2)	5.9 (1.5)	6.2 (1.3)	6.1 (1.3)	5.8 (1.5)
Meaning-3	6.3 (1.2)	6.2 (1.2)	6.2 (1.2)	6.4 (1.0)	6.3 (1.0)	5.9 (1.4)
Competence-1	5.3 (1.6)	5.1 (1.8)	5.2 (1.7)	5.6 (1.4)	5.5 (1.4)	5.3 (1.7)
Competence-2	4.5 (1.8)	4.4 (2.0)	4.6 (1.9)	5.0 (1.6)	4.8 (1.8)	4.8 (1.9)
Competence-3	5.3 (1.8)	5.0 (1.9)	5.7 (1.3)	5.3 (1.4)	4.9 (1.5)	5.5 (1.4)
Self-determination-1	4.3 (1.9)	4.2 (1.9)	4.6 (2.0)	4.7 (1.7)	4.9 (1.6)	5.1 (1.8)
Self-determination-2	3.5 (2.3)	3.5 (1.9)	4.2 (2.2)	3.7 (1.9)	4.1 (2.0)	4.5 (2.0)
Self-determination-3	4.0 (2.3)	4.0 (1.9)	4.1 (2.0)	4.1 (2.0)	4.5 (1.9)	4.5 (2.0)
Impact-1	4.4 (2.0)	4.4 (1.9)	4.2 (2.2)	5.3 (1.4)	5.1 (1.7)	4.8 (1.8)
Impact-2	3.5 (1.8)	3.6 (2.0)	3.8 (2.1)	4.2 (1.7)	4.5 (1.7)	4.2 (1.8)
Impact-3	3.7 (2.0)	4.3 (2.0)	4.0 (1.9)	4.5 (1.8)	5.0 (1.5)	4.1 (1.9)

^a ^Three items compose each empowerment construct.

Regarding usage of the application, the mean number of visits as recorded by a log file analyzer was 13.27 (SD 6.68). The mean time spend on the website was 4.8 (SD 3.2) minutes per visit. Table 3 reports some usage data related to the main features implemented in the experimental conditions. Given that these figures suggest that the application was not used very often, in a preliminary phase of the analysis, we introduced the number of visits and the time spent on the application as potential moderators of the relationships implied by the theoretical model. For the sake of brevity, we cannot report the whole preliminary evaluation, but the main result was that, at the observed level of usage, we found no significant moderating effect. Although this result does not exclude the presence of a trend (ie, that running the study for a longer period would generate significant interaction effects), it suggests that usage of the application had no strong influence on the relationships investigated in the present analysis. It must be acknowledged, however, that the scant usage of the system may understate the results of the present analysis. Under the assumption that more frequent usage should lead to larger effects, the differential impact of some features that were seldom used (eg, the chat room) might stay covered. We accounted for this in the power and effect size considerations that are detailed in Multimedia Appendix 1 [78-82].

**Table 3:** Usage data (mean, SD) across experimental conditions.

Variable	Group 1	Group 2	Group 3
Time per visit (minutes)	4.2 (10.2)	4.8 (16.4)	5.4 (13.2)
Number of visits to library	8.13 (3.5)	NA^a^	7.6 (3.5)
Number of visits to virtual gymnasium	9.0 (3.1)	NA	10.2 (0.6)
Number of visits to testimonials	12.9 (2.5)	NA	11.4 (1.2)
Number of visits to Web forum	NA	10.5 (22.1)	8.5 (8.1)
Number of visits to chat room	NA	2.1 (1.3)	1.8 (4.3)

^a ^Not applicable.

### Testing Hypotheses

Our hypotheses detailed the role of knowledge and patient empowerment as mediating variables of a possible effect of the availability of interactive elements in an eHealth intervention. Results on the differences between the three study groups indicate no significant difference in fibromyalgia impact (*F*_2,148 _= 0.824, *P *= .44). This means that the two groups who were offered interactive elements did not benefit more from the website than the group offered only static elements. The absence of a difference renders the question of mediating variables obsolete. However, we did compute the model (and report it here) to determine at what stage the hypothesized relationships failed to emerge.

**Figure 3:**
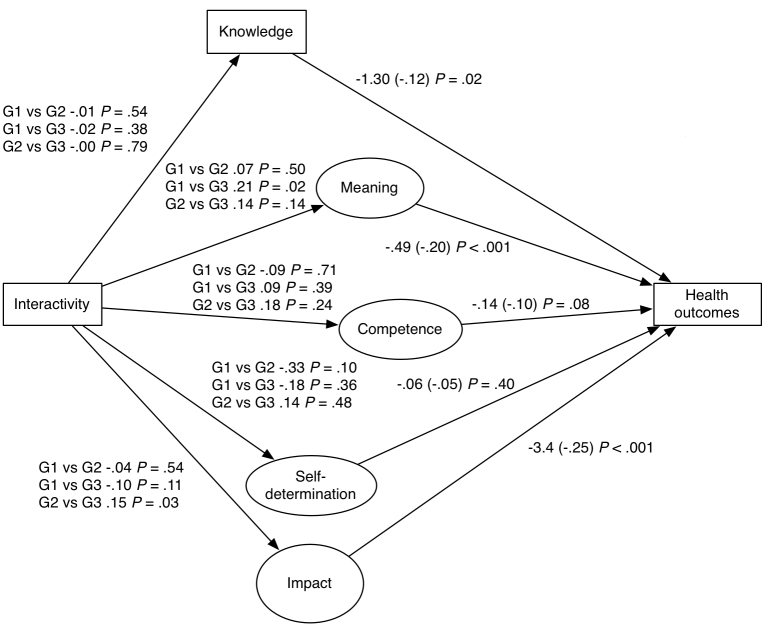
Overall model results. G1 = static-only study group; G2 = interactive-only study group; G3 = full application study group. Standardized coefficients are in parentheses (see Multimedia Appendix 2-7 for details).

The following five steps address one of the five focal dependent variables (knowledge, and the four dimensions of empowerment). Each section first reports the results of the effects of one of the focal dependent variables on the endogenous variable, health outcomes (operationalized as patient assessment of fibromyalgia impact), and then the effects of the main independent variable on the focal dependent variable. This means that hypotheses 1 and 6 are treated in one step, as are hypotheses 2 and 7, 3 and 8, an so on. To make the presentation clearer, we summarize the results in Figure 3. All five models are available in Multimedia Appendix 2, Multimedia Appendix 3, Multimedia Appendix 4, Multimedia Appendix 5, and Multimedia Appendix 6. The five steps do not mention the autoregressive effects of the focal dependent variables at time 1 on time 2 and the endogenous variable at time 1 on time 2. All these effects were highly significant.

### The Role of Knowledge

As predicted by the hypothesized model (hypothesis 6), *knowledge *at posttest significantly predicted health outcomes at posttest. On average, for every 1 unit that knowledge increased, negative health outcomes were predicted to decrease by 1.3 units, everything else being equal. We further hypothesized that interactivity enabled in the application increases patients’ knowledge (hypothesis 1). This result should reflect a significant difference between groups 2 and 3 (higher score) and group 1 (lower score). Despite the significant effect of knowledge on patient assessment of fibromyalgia impact, interactivity did not have any impact on knowledge, as the absence of significant differences between the study groups testifies. Given this result, hypothesis 1 was not confirmed.

### The Role of Meaning

As predicted by the hypothesized model (hypothesis 7), *meaning * at posttest significantly predicted assessment of impact at posttest. On average, for every 1 unit that meaning increased, negative assessments were predicted to decrease by 0.49 units, everything else being equal. We further hypothesized that interactivity enabled in the application should increase the patients’ meaning score (hypothesis 2). There should be a significant difference between the groups with (higher score) and without (lower score) interactivity. The only direct effect of functional interactivity on meaning can be observed in the significant difference between group 1 (ie, patients provided with the static version of the tool) and group 3 (ie, patients provided with the full-fledged application). However, the direction of the effect is not in as expected. Patients who had access to both static and interactive elements on the site scored significantly lower in meaning than did people with the static version only. Therefore, hypothesis 2 was not supported.

### The Role of Competence

Contrary to the eHealth effect model prediction (hypothesis 8), *competence *at posttest did not significantly predict patient assessment of fibromyalgia impact at posttest. We also hypothesized that the interactivity enabled in the application should increase the patients’ competence score (hypothesis 3). However, interactivity did not have any such impact on competence, as testified by the absence of significant differences between the study groups. Given this result, hypothesis 3 was not confirmed.

### The Role of Self-determination

Contrary to the eHealth effect model prediction, *self-determination *at posttest did not significantly predict patient assessment of fibromyalgia impact at posttest. Thus, hypothesis 9 was not supported. We also hypothesized that the interactivity enabled in the application should increase the patients’ self-determination score (hypothesis 4). No significant direct effect of functional interactivity was observed, as indicated by the absence of significant differences between the study groups. Therefore, hypothesis 4 was not confirmed.

### The Role of Impact

In accordance with the eHealth effect model prediction (hypothesis 10), *impact *at posttest significantly predicted health outcomes at posttest. The impact of the syndrome as perceived by the patient appears to make the largest difference in health outcomes. From this experimental data, we observed that for every 1 unit impact score increased, negative health outcomes were predicted to decrease on average by 3.4 units (or a quarter of a standard deviation), which is indeed a relevant finding. Given this result, hypothesis 10 was confirmed. We also hypothesized that the interactivity enabled in the application should increase the patients’ impact score (hypothesis 5). This result should reflect a significant difference between groups 2 and 3 (higher scores) and group 1 (lower score). The only significant difference, though, was found between groups 2 and 3. Patients provided with the interactive-only application (group 2) scored significantly higher on impact than did patients who had access to both the interactive and the static elements (group 3). Hypothesis 5, consequently, was not confirmed.

Although not of primary relevance in this analysis, it is also interesting that the number of years that patients had FMS influenced their impact score. On average, for every 1 additional year of illness experience, the patients’ impact score was predicted to decrease by 0.04 units. The total effect of years of illness experience on health outcomes was 0.07, meaning that an additional year of FMS on average increased negative health outcomes by 0.07 units, and did so by decreasing the impact that patients perceived the syndrome to have on them. In other words, the lower a patient’s experienced control over the syndrome (impact score), the worse the health outcomes score will be.

## Discussion

In summary, the presence of interactive elements in our eHealth intervention did not affect knowledge, did not affect patient empowerment in the expected direction (but reduced the empowerment dimension of meaning), and did not improve the health outcome of perceived fibromyalgia impact. In contrast to other studies, ours did not find beneficial effects of functional interactivity. However, knowledge and two dimensions of empowerment (meaning and self-determination) did affect health outcomes. Overall, the experimental findings suggest that only some of the hypothesized relationships held true (see Figure 3 and Multimedia Appendix 7).

The strongest relationships concern the effects of knowledge, meaning, and impact on health outcomes. These results show that cognition and empowerment, at least in some of its dimensions, are strong predictors of health outcomes, and eHealth applications should try to target and enhance these individual characteristics.

The relationships between functional interactivity and knowledge, empowerment, and health outcomes was not supported. Indeed, functional interactivity had an impact on two empowerment dimensions (meaning and impact), but in both cases the effect was not in line with the original predictions. In all cases, patients provided with the less complete version of the eHealth application reported higher scores on these dimensions of empowerment than did those exposed to the full application. This unexpected result may be due to the possibility that viewing static information helped individuals in group 1 make more sense of their condition than did a combination of static and interactive features. An alternative explanation is that patients provided with the full-featured application were overloaded by the functions and this badly affected their ability to understand their health condition.

Both unexpected results may also be unintended consequences of item wording. The empowerment dimension of impact was measured with items that varied the notion of control and influence over a person’s quality of life and health status in general, while all items used for the other three dimensions specifically referred to FMS. Adding interactive to static features may have increased (or, given the other results, more likely not affected) a sense of impact related specifically to FMS (which was hypothesized), but at the same time reduced a sense of general impact (which is closer to what was actually measured). This highlights the general possibility that condition-specific eHealth applications, along with their intended effects on condition-specific empowerment, may have adverse effects on a general sense of empowerment. Or, in plain words: patients who are effectively being told their condition is manageable and controllable may get the impression that other conditions are not. This has implications for the conceptualization and measurement of empowerment as well as for the design of eHealth applications.

The empowerment dimension of meaning was measured with items varying the notion of the importance of coping with FMS. The finding that the scores decreased when interactive features were added to static ones can be interpreted in two ways, depending on how respondents may have understood the term *importance*. If importance was understood as relevance, the unexpected finding could be interpreted as an undesirable distraction from actually important matters, caused by the interactive elements. If, however, importance was understood as urgency, the result could indicate a desirable effect: as patients improved their ways of coping (not least by the eHealth application), the subject of coping became less urgent to think about. Methodologically, this would imply that the measure used for this dimension needs to be revised.

The relationships between interactivity and knowledge, self-determination, or competence were not confirmed. Regarding knowledge, a plausible explanation is that the patients were generally knowledgeable about the condition even before the pretest (indeed, the mean score at pretest was 0.60, SD 0.2). Because of this, the application did not make a significant difference with regard to this construct. The same holds true for competence, for which initial score was relatively high across the study groups (5.25, SD 1.7). Regarding self-determination, at least two interpretations seem reasonable. First, while self-determination is a construct referring to individuals’ autonomy, the application was not meant to increase independence in facing the condition, but rather to support embeddedness within a community of patients. Second, interactivity is only one of the factors that may contribute to increase the overall sense of autonomy of a patient with a chronic condition. Although it is theoretically reasonable to include this dimension as part of empowerment, the specific context of investigation may not fully reflect its relevance. Further theoretical work is needed to expand this issue.

It is difficult to relate these results to the empirical literature, which is scarce. Empirical studies that are concerned with interactivity in health applications, if they exist at all, address the effects on quite different dependent variables, such as attitudes to and satisfaction with the application [53], efficiency of professional learning [54], risk assessments [56], and, for political campaign websites, amount of time spent on the site and content recall [73]. Warnick et al [73] pointed out, quite in line with our results, that too much interactivity can decrease content recall. Neither empowerment nor health outcomes have, to our knowledge, been empirically related to interactivity, and of course the application we used has not been studied, nor has our measure of empowerment been used, as it was developed for this study. Hopeful expectations for a beneficial impact of eHealth interactivity (eg, [10]) should be tempered unless other empirical research supports them.

Generally, support for the mediator perspective on interactivity effects (with knowledge and empowerment functioning as mediators) was weak because the hypothesized effects of interactivity on knowledge and empowerment could not be established.

### Study Limitations

Some limitations of this study should be acknowledged. In respect to interactivity, the preferred approach mostly relied on the functional perspective [18]. This choice allowed testing for the different enabling functions implemented in the eHealth intervention. However, the dimension of perceived interactivity [15,17] may play a major role as well. In this study, we assumed that the chosen functions had a different degree of interactivity without considering whether the patients perceived this difference. Ideally, both functional and perceived interactivity must be considered in an integrated and holistic model, as shown in a recent contribution to the study of this construct [83].

The construct of knowledge was largely based on the critical and integrative perspective on health literacy. However, the aggregate average score provided by the knowledge test may not fully capture its theoretical complexity. For example, in Schulz and Nakamoto’s conception, individual judgment skills should ideally be included in a full measure of integrative health literacy [26].

Regarding our methods, an objection can be advanced for the lack of a pure control group in the study phase. Patients provided with the static version of the application were used as a baseline, but we did not include patients who were not exposed to the application. This limitation was mainly linked to feasibility issues in reaching a sufficiently large number of FMS patients. In addition, it is not uncommon to provide people in the control group with some kind of neutral intervention, and this principle was applied in the study.

### Implications and Future Directions

Despite these limitations, this study provided interesting insights into the differential effect of enabling functions implemented in eHealth interventions, providing some evidence of the impact of interactivity in the health context.

First, the combination of interactive and static features is not necessarily a turnkey solution to enhance patients’ knowledge and empowerment. For example, the presence of interactive features did not significantly improve the individual level of knowledge. When tested as stand-alone feature, however [84], the library of information as implemented in ONESELF had an impact on patients’ knowledge. Given that we measured knowledge in relation to FMS, however, this result is hardly generalizable to other health conditions. More holistic and comprehensive measures of individual knowledge would help in comparing results on this construct.

A second consideration relates to other studies [56,70,73], which found that interactivity can enhance individual efficacy and self-determination. The present study rejected this claim by testing the effects of this construct on a more holistic conceptualization of empowerment.

A third, more methodological observation is that testing individual features separately or grouped into clusters decreases the effect size of the overall application. The present analysis is not suggesting that one should limit an eHealth intervention to some functions, but rather that the choice of these features should be theory or goal driven. This is even more important considering that our results showed that the effect of the full-fledged application was generally weaker than a more focused version. It is possible that patients provided with the more complex application needed more time before mastering the system and the impact was somehow delayed. A longitudinal evaluation may provide further insights into this hypothesis. Indeed, the stability of a certain effect is rarely tested in eHealth studies [85]. This is partly due to the costs associated with longitudinal evaluations and partly linked to the high level of attrition [82] between pretest and posttest.

At the time of writing this paper, research on health communication and the Internet is rapidly evolving. While the debate on the theories and constructs that must be considered in the design and evaluation of eHealth applications is lively, several efforts are being made to define standard procedures to conduct and report empirical studies on eHealth interventions [86]. In line with the scientific debate around eHealth, an underlying theme reappeared throughout this work: the need to integrate a theory-driven approach into eHealth research. The model of eHealth effects defined and tested in this study is a first step on this route, but is far from complete, especially as the model did not hold. Future directions of this endeavor should focus on adapting, complementing, and refining the model and its evaluation. The constructs included in the model of eHealth effects posited some limitations at the theoretical level. For example, the construct of interactivity should be refined to include both functional and perceived perspectives. Although Rafaeli and Ariel [15] argued that interactivity is primarily a subjective construct, exclusion of interactivity at the functional level would not provide any insight into the mechanisms that occur within eHealth applications. A more integrative perspective such as the one recently proposed by Yoo [83], who combined medium and audience interactivity, is preferable and should be pursued to refine the model.

Along the same lines, while the general validity of the model of empowerment as proposed by Thomas and Velthouse [40] and Spreitzer [75] was assessed in the health care setting, other models of empowerment (eg, [87]) might be considered and integrated to develop an improved measure of this construct.

In general terms, a model should be as predictive and explanatory as it is parsimonious. Without overexpanding the eHealth effects model, there are some constructs that should be considered for its improvement.

The first two of these constructs relate to the literacy and knowledge dimensions. According to Schulz and Nakamoto [26], the construct of judgment skills as the ability to integrate procedural and declarative knowledge into one’s own experience should be included in the model to complement the construct of knowledge. Before integrating the judgment dimension, however, further effort should be put into translating it into operational measures. Additionally, the construct of media literacy [88] can further improve the cognitive component of the model. Rosenbaum and colleagues [89] defined media literacy as the understanding of the influences that occur between the media, producers, and users. Thus, media literacy can be considered a mediator between exposure to a certain medium and its effects on opinions and behavior. As such, together with health literacy, it can make a significant difference in the effects of eHealth interventions.

Eventually, an important construct that should be included in the model is computer self-efficacy, or one’s confidence in using a computer and gaining benefits from it [90,91]. Studies on eHealth often have generalizability issues because the reference population is composed of people who are implicitly considered skilled in using a computer. The CONSORT-EHEALTH guidelines for conducting research on eHealth stress the need to avoid this assumption and urge systematically testing individuals’ ability and confidence in using technology [86]. The construct of computer self-efficacy and its measurement can serve to control for this aspect, while providing further insights into the mechanisms that determine the effectiveness of eHealth interventions.

## Multimedia Appendix 1

CONSORT-EHEALTH Checklist V 1.6.

## Multimedia Appendix 2

Model of the effect of interactivity on knowledge.

## Multimedia Appendix 3

Model of the effect of interactivity on meaning.

## Multimedia Appendix 4

Model of the effect of interactivity on competence.

## Multimedia Appendix 5

Model of the effect of interactivity on self-determination.

## Multimedia Appendix 6

Model of the effect of interactivity on impact.

## Multimedia Appendix 7

Summary of hypotheses tested and subsequent results.

## Multimedia Appendix 8

CONSORT-EHEALTH Checklist V 1.6 [92].

## Abbreviations

FAS: Fibromyalgia Assessment Status
FIQ: Fibromyalgia Impact Questionnaire
FMS: fibromyalgia syndrome
