# Supplementary material for: Effects of Functional Interactivity on Patients’ Knowledge, Empowerment, and Health Outcomes: An Experimental Model-Driven Evaluation of a Web-Based Intervention
Source: J Med Internet Res. 2012 Jul 18;14(4):e105. doi: 10.2196/jmir.1953 (PMC3409610; doi:10.2196/jmir.1953)
Supplement: Supplementary file 1 [file jmir_v14i4e105_app1.pdf]

MULTIMEDIA APPENDIX 1

Structural Equation Modeling: summary of preliminary analyses

Statistical power, outliers, normality, and missing data

To determine the appropriate sample size, SEM requires that statistical power together with issues of the stability of the covariance matrix and the use of asymptotic theory be taken into account [78, 79]. A power analysis was conducted for a path coefficient for a predictor that accounts for at least 5% unique variance in the outcome. Two models have been tested (one with a maximum number of 8 predictors for a linear equation, and the other with a maximum number of 5 predictors), and the analysis was replicated for both scenarios. A square multiple correlation of 0.30, a 0.05 alpha level, and a two-tailed test were assumed. The sample size of 165 yielded power of .99 for linear models with either 8 or 5 predictors.

Each continuous variable was evaluated for outliers by examining its frequency distribution at the univariate level to identify scenarios where extreme scores occurred for a small number of respondents. No univariate outliers were evident on any of the variables included in the models. Multivariate outliers analysis was pursued using model based and non-model based techniques [80]. Both techniques revealed no evident outliers.

Multivariate normality was tested using Mardia's test for each model analyzed. All tests yielded statistically significant results, suggesting the presence of non-normality at the multivariate level. Univariate normality was assessed for each continuous variable using skewness and kurtosis indices. Troublesome skewness and kurtosis values are evident for the measure of the meaning dimension of empowerment (both at baseline and at the post-test). Given this, the decision was made to pursue parameter estimation using bootstrapping with 2,000 replications as implemented in AMOS® 18. Given the use of bootstrapping, the p-value for overall fit of the tested models was calculated using the Bollen-Stine bootstrap approach in place of the traditional Chi-square statistic [81].

For what concerns missing data, a total of 14 individuals (8.4%) did not complete the second assessment and their values are missing on all variables. The total number of non-respondents was evenly distributed across the experimental conditions, as shown in Figure 2. An attrition rate of 8.4% can be considered small in magnitude for eHealth experiments [82]. However, in order to check for any potential bias, a dummy variable was created to discriminate individuals who completed both assessments and the ones who failed to complete the post-test. This dummy variable was correlated with all other variables in the model measured in the pre-test. The associations were all statistically non-significant, suggesting that post-test participants do not significantly differ from dropouts. Given these results, the Expectation Maximization (EM) algorithm implemented in SPSS® 17 was used to impute the values for the missing cases.
