# Supplementary material for: Effects of Functional Interactivity on Patients’ Knowledge, Empowerment, and Health Outcomes: An Experimental Model-Driven Evaluation of a Web-Based Intervention
Source: J Med Internet Res. 2012 Jul 18;14(4):e105. doi: 10.2196/jmir.1953 (PMC3409610; doi:10.2196/jmir.1953)
Supplement: Supplementary file 2 [file jmir_v14i4e105_app2.pdf]

MULTIMEDIA APPENDIX 2

Model of the effect of interactivity on knowledge.

**Endogenous variable**	*Standardized Disturbance*	*Explained Variance (R^2)*
Knowledge T2	.52	.48
HO T2	.49	.51

Structural model

Effects	*B*	*P value*	*B 95% CI*	*b*
Knowledge T1 to Knowledge T2	.65	< .001	.52 to .76	.69
Age to Knowledge T2	.00	.84	-.002 to .002	-.01
YD to Knowledge T2	-.00	.44	-.005 to .002	-.04
HO T1 to HO T2	.68	< .001	.57 to .79	.69
Knowledge T2 to HO T2	-1.3	.02	-2.4 to -0.2	-.12
Mean differences				
G1 vs. G2	-.01	.54	-0.6 to .03	-
G1 vs. G3	-.02	.38	-.07 to .03	-
G2 vs. G3	-.00	.79	-.05 to .00	-

Notes:

T1 = pre-test, T2 = post-test, YD = years since first diagnosis, HO = health outcomes, G1/G2/G3 = experimental groups, B = unstandardized coefficient, b = standardized coefficient, CI = confidence interval.

Bollen-Stine p-value = .993; CFI = 1; RMSEA = 0; p-value for close fit = .995; Standardized RMR = .007. No theoretically meaningful modification indices > 4 and no values > 1.96 in the standardized residuals covariance matrix.
