# Supplementary material for: Effects of Functional Interactivity on Patients’ Knowledge, Empowerment, and Health Outcomes: An Experimental Model-Driven Evaluation of a Web-Based Intervention
Source: J Med Internet Res. 2012 Jul 18;14(4):e105. doi: 10.2196/jmir.1953 (PMC3409610; doi:10.2196/jmir.1953)
Supplement: Supplementary file 3 [file jmir_v14i4e105_app3.pdf]

MULTIMEDIA APPENDIX 3

Model of the effect of interactivity on meaning.

**Endogenous variable**	*Standardized Disturbance*		*Explained Variance (R^2)*	
Meaning T2	.48		.52	
HO T2	.47		.53	
**Measurement model**	*Loading*		*Reliability*	
L Meaning T1 to Item1 T1	.78		.60	
L Meaning T1 to Item2 T1	.85		.72	
L Meaning T1 to Item3 T1	.82		.67	
L Meaning T2 to Item1 T2	.70		.50	
L Meaning T2 to Item2 T2	.88		.77	
L Meaning T2 to Item3 T2	.87		.75	
**Structural model**				
Effects	*B*	*P value*	*B 95% CI*	*b*
Meaning T1 to Meaning T2	.53	< .001	.31 to .79	.67
Age to Meaning T2	-.009	.06	-.02 to .002	-.12
YD to Meaning T2	-.007	.43	-.04 to .12	-.04
HO T1 to HO T2	.68	< .001	.58 to .78	.69
Meaning T2 to HO T2	-.49	< .001	-.91 to -.22	-.20
Mean differences				
G1 vs. G2	.07	.50	-.12 to .37	-
G1 vs. G3	.21	.02	.01 to .48	-
G2 vs. G3	.14	.14	-.14 to .40	-

Notes:

PT = product-term, T1 = pre-test, T2 = post-test, YD = years since first diagnosis, HO = health outcomes, G1/G2/G3 = experimental groups, B = unstandardized coefficient, b = standardized coefficient, CI = confidence interval.

Bollen-Stine p-value = .618; CFI = .99; RMSEA = .035; p-value for close fit = .738; Standardized RMR = .032. No theoretically meaningful modification indices > 4 and no values > 1.96 in the standardized residuals covariance matrix.
