# Supplementary material for: Effects of Functional Interactivity on Patients’ Knowledge, Empowerment, and Health Outcomes: An Experimental Model-Driven Evaluation of a Web-Based Intervention
Source: J Med Internet Res. 2012 Jul 18;14(4):e105. doi: 10.2196/jmir.1953 (PMC3409610; doi:10.2196/jmir.1953)
Supplement: Supplementary file 4 [file jmir_v14i4e105_app4.pdf]

MULTIMEDIA APPENDIX 4

Model of the effect of interactivity on competence.

**Endogenous variable**	*Standardized Disturbance*		*Explained Variance (R^2)*	
Competence T2	.51		.49	
HO T2	.50		.50	
**Measurement model**	*Loading*		*Reliability*	
L Competence T1 to Item1 T1	.85		.72	
L Competence T1 to Item2 T1	.89		.79	
L Competence T1 to Item3 T1	.57		.32	
L Competence T2 to Item1 T2	.86		.73	
L Competence T2 to Item2 T2	.93		.86	
L Competence T2 to Item3 T2	.61		.37	
**Structural model**				
Effects	*B*	*P value*	*B 95% CI*	*b*
Competence T1 to Competence T2	.61	< .001	.44 to .77	.68
Age to Competence T2	-.016	.06	-.03 to .007	-.11
YD to Competence T2	-.02	.10	-.06 to .01	-.10
HO T1 to HO T2	.67	< .001	.56 to .78	.68
Competence T2 to HO T2	-.14	.08	-.29 to .02	-.10
Mean differences				
G1 vs. G2	-.09	.71	-.50 to .42	-
G1 vs. G3	.09	.39	-.27 to .47	-
G2 vs. G3	.18	.24	-.27 to .60	-

Notes:

PT = product-term, T1 = pre-test, T2 = post-test, YD = years since first diagnosis, HO = health outcomes, G1/G2/G3 = experimental groups, B = unstandardized coefficient, b = standardized coefficient, CI = confidence interval.

Bollen-Stine p-value = .09; CFI = .978; RMSEA = .051; p-value for close fit = .445; Standardized RMR = .043. No theoretically meaningful modification indices > 4 and no values > 1.96 in the standardized residuals covariance matrix.
