# Supplementary material for: Effects of Functional Interactivity on Patients’ Knowledge, Empowerment, and Health Outcomes: An Experimental Model-Driven Evaluation of a Web-Based Intervention
Source: J Med Internet Res. 2012 Jul 18;14(4):e105. doi: 10.2196/jmir.1953 (PMC3409610; doi:10.2196/jmir.1953)
Supplement: Supplementary file 5 [file jmir_v14i4e105_app5.pdf]

MULTIMEDIA APPENDIX 5

Model of the effect of interactivity on self-determination.

**Endogenous variable**	*Standardized Disturbance*	*Explained Variance (R^2)*
Self-determination T2	.48	.52
HO T2	.51	.49

**Measurement model**	*Loading*	*Reliability*
L Self-determination T1 to Item1 T1	.75	.56
L Self-determination T1 to Item2 T1	.89	.79
L Self-determination T1 to Item3 T1	.80	.64
L Self-determination T2 to Item1 T2	.76	.57
L Self-determination T2 to Item2 T2	.91	.82
L Self-determination T2 to Item3 T2	.75	.56

Structural model

Effects	*B*	*P value*	*B 95% CI*	*b*
Self-det. T1 to Self-det. T2	.61	< .001	.47 to .78	.70
Age to Self-determination T2	-.01	.14	-.005 to .03	-.09
YD to Self-determination T2	-.003	.86	-.03 to .03	-.01
HO T1 to HO T2	.68	< .001	.56 to .80	.69
Self-determination T2 to HO T2	-.06	.40	-.23 to .10	-.05
Mean differences				
G1 vs. G2	-.33	.10	-.73 to .04	-
G1 vs. G3	-.18	.36	-.63 to .23	-
G2 vs. G3	.14	.48	-.28 to .57	-

Notes:

T1 = pre-test, T2 = post-test, YD = years since first diagnosis, HO = health outcomes, G1/G2/G3 = experimental groups, B = unstandardized coefficient, b = standardized coefficient, CI = confidence interval.

Bollen-Stine p-value = .09; CFI = .978; RMSEA = .053; p-value for close fit = .415; Standardized RMR = .046. No theoretically meaningful modification indices > 4 and no values > 1.96 in the standardized residuals covariance matrix.
