# Supplementary material for: Effects of Functional Interactivity on Patients’ Knowledge, Empowerment, and Health Outcomes: An Experimental Model-Driven Evaluation of a Web-Based Intervention
Source: J Med Internet Res. 2012 Jul 18;14(4):e105. doi: 10.2196/jmir.1953 (PMC3409610; doi:10.2196/jmir.1953)
Supplement: Supplementary file 6 [file jmir_v14i4e105_app6.pdf]

MULTIMEDIA APPENDIX 6

Model of the effect of interactivity on impact.

**Endogenous variable**	*Standardized Disturbance*		*Explained Variance (R^2)*	
Impact T2	.44		.56	
HO T2	.47		.53	
**Measurement model**	*Loading*		*Reliability*	
L Impact T1 to Item1 T1	.83		.68	
L Impact T1 to Item2 T1	.82		.67	
L ImpactT1 to Item3 T1	.93		.86	
L Impact T2 to Item1 T2	.80		.64	
L Impact T2 to Item2 T2	.83		.68	
L Impact T2 to Item3 T2	.89		.79	
**Structural model**				
Effects	*B*	*P value*	*B 95% CI*	*b*
Impact T1 to Impact T2	.53	< .001	.41 to .66	.71
Age to Impact T2	-.007	.34	-.02 to .008	-.05
YD to Impact T2	-.04	.007	-.08 to -.004	-.16
HO T1 to HO T2	.60	< .001	.48 to .72	.62
Impact T2 to HO T2	-3.4	< .001	-5.2 to -1.8	-.25
Mean differences				
G1 vs. G2	-.04	.54	-.18 to .10	-
G1 vs. G3	-.10	.11	-.03 to .25	-
G2 vs. G3	.15	.03	.009 to .27	-

Notes:

T1 = pre-test, T2 = post-test, YD = years since first diagnosis, HO = health outcomes, G1/G2/G3 = experimental groups, B = unstandardized coefficient, b = standardized coefficient, CI = confidence interval.

Bollen-Stine p-value = .19; CFI = .967; RMSEA = .071; p-value for close fit = .098; Standardized RMR = .044. No theoretically meaningful modification indices > 4 and no values > 1.96 in the standardized residuals covariance matrix.
