# Supplementary material for: Effects of Functional Interactivity on Patients’ Knowledge, Empowerment, and Health Outcomes: An Experimental Model-Driven Evaluation of a Web-Based Intervention
Source: J Med Internet Res. 2012 Jul 18;14(4):e105. doi: 10.2196/jmir.1953 (PMC3409610; doi:10.2196/jmir.1953)
Supplement: Supplementary file 7 [file jmir_v14i4e105_app7.pdf]

MULTIMEDIA APPENDIX 7

Summary of hypotheses tested and subsequent results.

**Hypothesis**	**Result**	**Comment**
H1: Patients who used an application that offered interactive functions will become more knowledgeable than patients not offered this function.	Not confirmed	No effect of the different enabling features was found on knowledge.
H2: Patients who used an application that offered interactive functions will achieve a higher score on the empowerment dimension of meaning than patients not offered this function.	Not confirmed	There is an effect of the different enabling features on the meaning score, but not in the predicted direction. People in G3 scored significantly lower in meaning than people with the static version of the application.
H3: Patients who used an application that offered interactive functions will achieve a higher score on the empowerment dimension of competence than patients not offered this function.	Not confirmed	No effect of the different enabling features was found on the competence score.
H4: Patients who used an application that offered interactive functions will achieve a higher score on the empowerment dimension of self-determination than patients not offered this function.	Not confirmed	No effect of the different enabling features was found on the self-determination score.
H5: Patients who used an application that offered interactive functions will achieve a higher score on the empowerment dimension of impact than patients not offered this function.	Not confirmed	There is an effect of interactivity on the impact score, but people with the full-featured application scored lower on this outcome than people exposed to the interactive-only version of the intervention.
H6: The higher the knowledge of patients, the better (= lower) they assess their Fibromyalgia impact.	Confirmed	Individual level of knowledge is associated with improved health outcomes.
H7: The higher the meaning score of patients, the better they assess their Fibromyalgia impact.	Confirmed	Individual meaning score is associated with improved health outcomes.
H8: The higher the competence score of patients, the better they assess their Fibromyalgia impact.	Not confirmed	The empowerment dimension of competence did not have an effect on

		health outcomes.
H9: The higher the self-determination score of patients, the better they assess their Fibromyalgia impact.	Not confirmed	The empowerment dimension of self-determination did not have an effect on health outcomes.
H10: The higher the impact score of patients, the better they assess their Fibromyalgia impact.	Confirmed	The empowerment dimension of impact had a very strong effect on health outcomes in the predicted direction.
